# Neural Correlates of Antisocial Behavior: The Victim’s Perspective

**DOI:** 10.3390/brainsci13030474

**Published:** 2023-03-10

**Authors:** Nicolò Trevisan, Giulia Cattarinussi, Daniele Olivo, Andrea Di Ciano, Lucia Giudetti, Alan Pampallona, Katharina M. Kubera, Dusan Hirjak, Robert Christian Wolf, Fabio Sambataro

**Affiliations:** 1 Department of Neuroscience (DNS), University of Padua, 35121 Padua, Italy; 2 Padua Neuroscience Center, University of Padua, 35129 Padua, Italy; 3 Fondazione Giancarlo Quarta, 20129 Milan, Italy; 4 Center for Psychosocial Medicine, Department of General Psychiatry, Heidelberg University, 69115 Heidelberg, Germany; 5 Department of Psychiatry and Psychotherapy, Central Institute of Mental Health, Medical Faculty Mannheim, Heidelberg University, 68159 Mannheim, Germany

**Keywords:** antisocial behavior, self-centered behavior, social interaction, functional magnetic resonance imaging, brain activation, functional connectivity, temperament

## Abstract

Antisocial behavior involves actions that disregard the basic rights of others and may represent a threat to the social system. The neural processes associated with being subject to antisocial behavior, including social victimization, are still unknown. In this study, we used a social interaction task during functional magnetic resonance imaging to investigate the neural bases of social victimization. Brain activation and functional connectivity (FC) were estimated and correlated with the Big 5 Questionnaire, Temperament Evaluation in Memphis, Pisa and San Diego (TEMPS-M), and a Questionnaire of Daily Frustration scores. During social victimization, the right occipital and temporal cortex showed increased activation. The temporal cortex also had reduced FC with homotopic areas. Compared to the prosocial interaction, social victimization showed hyperactivation of the dorsomedial and lateral prefrontal cortex, putamen, and thalamus and increased FC of the medial-frontal–striatal–thalamic areas with the ventrolateral prefrontal cortex, insula, dorsal cingulate, and postcentral gyrus. Lastly, neuroticism, irritable temperament, and frustration scores were correlated with the magnitude of neural responses to social victimization. Our findings suggest that social victimization engages a set of regions associated with salience, emotional processing, and regulation, and these responses can be modulated by temperamental and personality traits.

## 1. Introduction

The human brain has an inherent expectation of access to social resources and social support being available, with responsive support being considered the default baseline [1]. Predictably, the affective impact of unresponsive support is much greater compared to responsive support, leading to short- and long-term distress, feelings of frustration, and altered mood states [2]. Social behaviors characterized by acting without taking into account the basic rights of others, which can result in a wide range of negative actions, are defined as antisocial behaviors [3]. Notably, antisocial behavior is not a pathological feature and does not imply a diagnosis of antisocial personality disorder. Antisocial behaviors include a variety of different conducts, such as ostracism or social exclusion. In particular, ostracism has emerged as a prominent area of inquiry because of its complex nature and profound impact on individuals’ psychological and social well-being. Ostracism is a specific type of subtle social exclusion that involves the intentional manipulation of personal relationships and social bonds to isolate or exclude individuals, resulting in them being left alone or ignored [4]. Ostracism can be a powerful tool for social control or punishment, and its effects can range from temporary hurt feelings to long-lasting psychological harm [4].

In social interactions, both the agent that performs the actions and the recipient, the person who is exposed to the actions, play a key role. Investigations in social psychology have highlighted the negative consequences of the perception of disrespect and ostracism for the recipient [5], leading to frustration and increased anger in people who experience these behaviors [6,7]. Indeed, the recipient of these behaviors can experience social victimization, a form of mistreatment that involves intentionally isolating and excluding individuals from social connections or being bullied by others. This type of victimization can cause significant distress to the victim and may be internalized in a way that is similar to the effects of physical victimization [8]. According to the temporal need-threat model of ostracism, the reaction of individuals exposed to social exclusion can be divided into three subsequent stages: immediate (or reflexive), coping (or reflective), and long-term (or resignation) [9]. If exposure to social exclusion persists, the individual’s resources to cope are weakened, leading to resignation, unworthiness, and depression [9]. To examine the psychological consequences of social exclusion, Williams et al. [10] introduced the cyberball paradigm, a virtual ball-tossing game in which the participant is excluded by the other two ostensible players. Interestingly, investigations employing the cyberball paradigm have shown that not receiving the ball or receiving it less frequently elicits negative affective reactions and threatens fundamental social needs [11]. The impact of social victimization varies between individuals, and this is due in part to differences in social responsiveness, which is the ability to understand, process, and react to social stimuli. Interestingly, social responsiveness is influenced by individual characteristics, which are either predominantly biology-based, such as temperament [12], or both biology- and environment-driven, such as personality [13]. Notably, a study using the cyberball paradigm showed that individuals with cluster A personality traits were buffered against ostracism’s negative impact on social pain, basic need satisfaction, and positive affect [14]. On the contrary, irritable temperament [15], defined as the tendency to experience excessive negative affect [16], has been associated with reactivity and negative interpretation in social contexts [17].

Recently, neuroimaging studies have begun to identify brain networks involved in social interactions. An investigation carried out by our research group demonstrated that practical help was associated with increased activation in a network of regions encompassing the bilateral superior temporal sulcus, the temporoparietal junction, the temporal pole, and the medial prefrontal cortex [18]. Regarding antisocial behaviors, a study evaluating the effect of violations of social expectations on brain activity reported activation of the temporoparietal junction and the medial prefrontal cortex, regions involved in empathy and mentalizing [19]. Furthermore, it has been shown that the so-called “pain matrix”, a neural network that is activated in the affective processing of physical and social pain and largely overlaps with the salience network, is recruited during social exclusion [20]. Furthermore, an fMRI investigation showed that the processing of social scenarios that violate social expectations was associated with increased fronto-insular activation, suggesting that the salience network was recruited when a departure from social norms was detected [21]. However, little is known about the mechanisms that support social victimization and how these responses could be related to intrinsic individual characteristics.

To this end, we designed a task that represented situations where an individual is exposed to antisocial behaviors using pictures and storytelling depicting real-life situations [22]. Notably, our task did not present the confounders of strategic components (multi-round economic games) and multiple confederates/players and was not limited to a specific social exclusion experience (cyberball). Additionally, to evaluate the effects of personality and temperament on neural responses to social interactions [14,23], we assessed personality traits with the Big Five Questionnaire (BFQ) [24] and affective temperaments with the Temperament Evaluation in Memphis, Pisa and San Diego (TEMPS-M) [25].

The overarching goal of this study was to elucidate the neural basis of social victimization in healthy subjects. Examining functional brain correlates of social victimization in healthy subjects is critical to shed light on mechanisms underlying the experience of antisocial behaviors without the bias of disease-specific processes, comorbid diagnoses, or medication. Here, we hypothesized that the neural processing of social victimization could follow the temporal need-threat model of ostracism. For this reason, we first tested the hypothesis that social victimization relative to a control condition would modulate the theory-of-mind (ToM) network, as well as the occipital cortex, as observed by Olivo et al. [18]. Second, we explored whether, compared to a prosocial interaction, social victimization would engage the brain regions associated with emotional processing, including the thalamus and basal ganglia, as well as those involved in the affective processing of pain and those implicated in emotional regulation, including cognitive appraisal and motor responses. Lastly, we posited that neural responses associated with social victimization would be correlated with personality traits associated with negative emotional processing, affective temperaments linked with impulsive/explosive responses, and negative reactions to frustrating events. Notably, a sample of young adults was chosen to maximize social responsiveness and relatively stable personality traits compared to other stages of life, where these factors may be not balanced [26].

## 2. Materials and Methods

### 2.1. Subjects

A total of 30 healthy young adults (17 females; age range 21–30 years) with normal or corrected-to-normal vision participated in the study. Exclusion criteria were (a) current or past medical, psychiatric, or neurological disease; (b) current or past substance use or dependence (excluding nicotine); (d) cognitive impairment; (e) head trauma with loss of consciousness; or (f) contraindications to magnetic resonance imaging. In addition, excessive head motion during the scan was another exclusion criterion from the analyses (see Supplementary Material). Written informed consent was obtained from all participants. The study was conducted in accordance with the principles expressed in the Declaration of Helsinki and was approved by the Institutional Review Board of the University of Parma (Italy, protocol ID: UNIPRMR750v1).

### 2.2. Social Interaction Task

**Figure 1:**
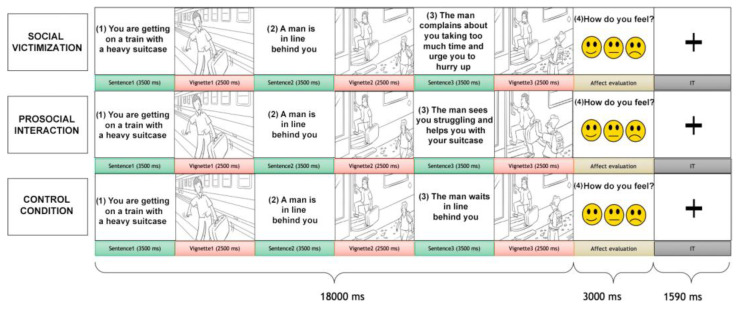
The social interaction task. Each trial comprised a social interaction condition that included an alternation of three sentences, three vignettes, and an affect-rating condition. The participant was requested to identify him-/herself with the receiver (the gray-haired character) who interacted with another person, the agent. Three conditions of social interaction were presented: social victimization, prosocial interaction, and (non-helping) control.

Participants were shown a sequence of three pictures alternated with three sentences, depicting a dyadic social interaction (Figure 1). The pictures included an interaction between two characters: the recipient, with whom the participants were requested to identify, and the agent, the person interacting with the recipient. One type of social interaction was displayed for each trial: social victimization, prosocial interaction, or control conditions. In the prosocial interaction, the recipient was helped to perform a task or given positive feedback by the agent. In the social victimization condition, the recipient was ignored during a task that needed external help or was given harsh feedback. The control condition showed a scene where the receiver accomplished a result without the direct involvement of the agent. Due to our focus on social victimization interactions, participants were shown 60 trials of social victimization, 30 of prosocial interaction, and 30 of the control condition without repetitions. After the presentation of the social interaction trial (18 s), an affect-rating image was presented for 3 s (see Supplementary Material).

### 2.3. Psychological Evaluation

Subjects performed a self-administered psychological evaluation before performing the task in the scanner. Personality traits were assessed with the BFQ [24] and temperament with the Italian version of the TEMPS-M [25]. Lastly, reactions to frustrating events were assessed using the Questionnaire of Daily Frustrations (QDF) [27] (see Supplementary Material).

### 2.4. Image Acquisition and Analysis

MRI scans were acquired on a 3 Tesla GE Discovery-MR750 scanner with a 16-channel multi-array head coil (see Supplementary Material). The social interaction task scan, which included the same number of trials per condition across 4 runs, comprised 338 volumes/run. Pre-processing was performed with SPM12 (see Supplementary Material). The task was modeled as a block design with four regressors for task conditions and one for the affective rating task and six nuisance motion parameters. During first-level analyses, whole-brain contrast maps of direct comparisons between social interaction conditions were calculated for each subject. Individual contrasts were then entered into a random-effects one-sample *t*-test analysis for each contrast. Psychophysiological interaction (PPI) analyses were performed to identify task-dependent changes in whole-brain connectivity with the clusters of increased activation during social victimization processing (see Supplementary Material). A model including the psychological regressors (t-contrasts for social victimization > neutral behavior and social victimization > prosocial interaction), the physiological regressors, and their interaction (cross-product of psychological and physiological regressors) was calculated for each subject. The positive and negative interaction terms were analyzed at the second level using one-sample *t*-tests. For all second-level analyses, we applied a *p* < 0.05 family-wise error correction at the cluster level with a cluster-defining threshold of uncorrected *p* < 0.001 at the voxel level.

### 2.5. Behavioral and Brain–Behavior Correlation Analyses

Reaction times were analyzed using repeated measures ANOVA between task conditions, followed by pairwise contrasts controlled for multiple comparisons with a Bonferroni correction. Unfortunately, due to a computer glitch, behavioral data from 15 participants were lost. Exploratory brain–behavior correlations between the first eigenvariate of each significant cluster from significant social victimization > prosocial interaction contrasts and psychological measures were performed using Spearman’s correlations, due to the non-normal distribution of the variables. For these analyses, the significance was set at *p* < 0.05. All analyses were performed with Jamovi 2.3.18.0 (see Supplementary Material).

## 3. Results

### 3.1. Behavioral Results

Repeated measures ANOVA showed a significant effect of the task condition on reaction times (*p* < 0.001), with social victimization having a faster response than the prosocial interaction and the control condition (*p* < 0.001) and the prosocial interaction having a faster response than the control condition (*p* < 0.001). Affective responses were congruent with the social interaction presented (see Table S1.)

### 3.2. Brain Activation

**Figure 2:**
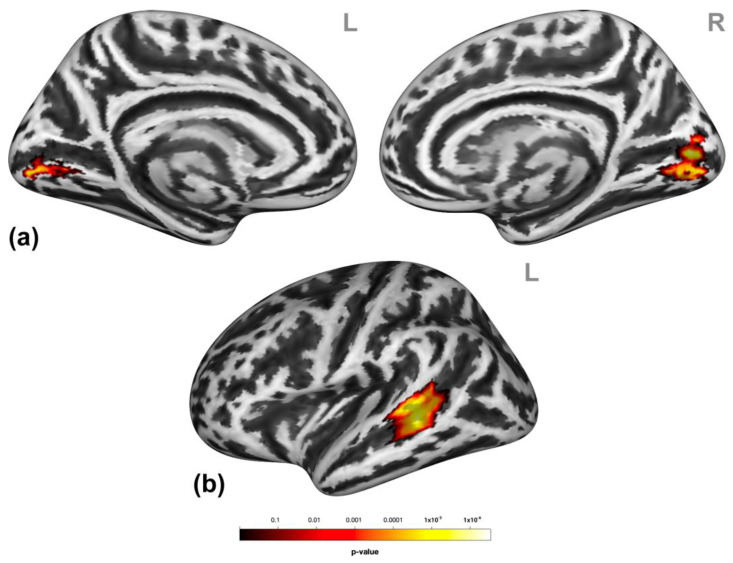
Neural effects of social victimization. Social victimization compared to the control condition was associated with increased activation in the occipital regions (**a**) and the left lateral middle and superior temporal gyri (MTG/STG) (**b**). Statistical maps are rendered on a Montreal Neurological Institute template with a cluster-level family-wise error correction of *p* < 0.05. The color bar indicates the *p*-value. L and R indicate the left and right brain hemispheres, respectively.

Participants showed significantly higher activation in the bilateral lingual gyrus and the left superior temporal gyrus/middle temporal gyrus (STG/MTG, Figure 2) during social victimization relative to the control condition.

**Figure 3:**
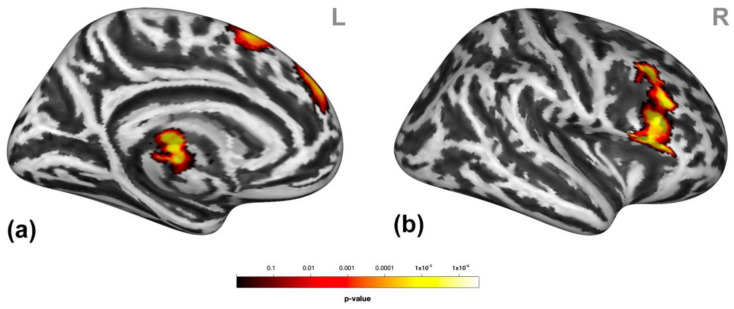
Neural response to social victimization compared to prosocial interaction. Social victimization compared to prosocial interaction was associated with increased activation in the medial/superior frontal gyrus, the pre-supplementary motor area, the thalamus (**a**), and the right inferior, middle, and superior frontal gyrus (**b**). Statistical maps are rendered on a Montreal Neurological Institute template with a cluster-level family-wise error correction of *p* < 0.05. The color bar indicates the *p*-value. L and R indicate the left and right brain hemispheres, respectively.

Participants presented higher activation in clusters encompassing the dorsomedial prefrontal cortex (dmPFC, entailing the medial frontal gyrus and superior frontal gyrus (mFG/SFG) and the bilateral pre-supplementary motor areas (SMA)), putamen, and thalamus and the right inferior frontal gyrus (IFG), middle frontal gyrus (MFG), and SFG (Figure 3) during social victimization compared to prosocial interaction. See Table S2 for detailed cluster information.

### 3.3. Psychophysiological Interactions

**Figure 4:**
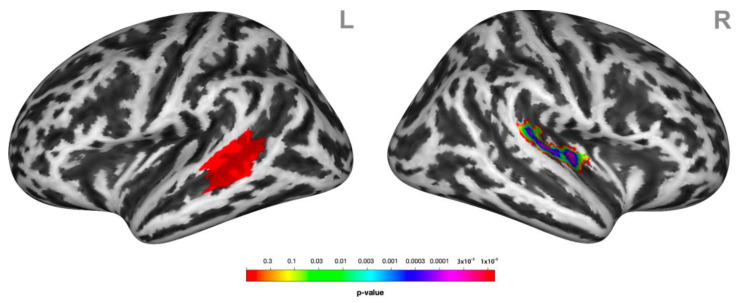
Psychophysiological interactions (PPIs) associated with receiving social victimization behavior. Functional coupling of the left lateral middle superior temporal gyrus (MTG/STG, here highlighted in red) (L) was decreased with the right superior temporal gyrus STG (R) during social victimization relative to the control condition. Statistical maps of the PPIs are rendered on a Montreal Neurological Institute template with a cluster-level family-wise error correction of *p* < 0.05. The color bar indicates the *p*-value. L and R indicate the left and right brain hemispheres, respectively.

During social victimization, the connectivity between the left STG/MTG and the contralateral STG was decreased relative to the control condition (Figure 4).

**Figure 5:**
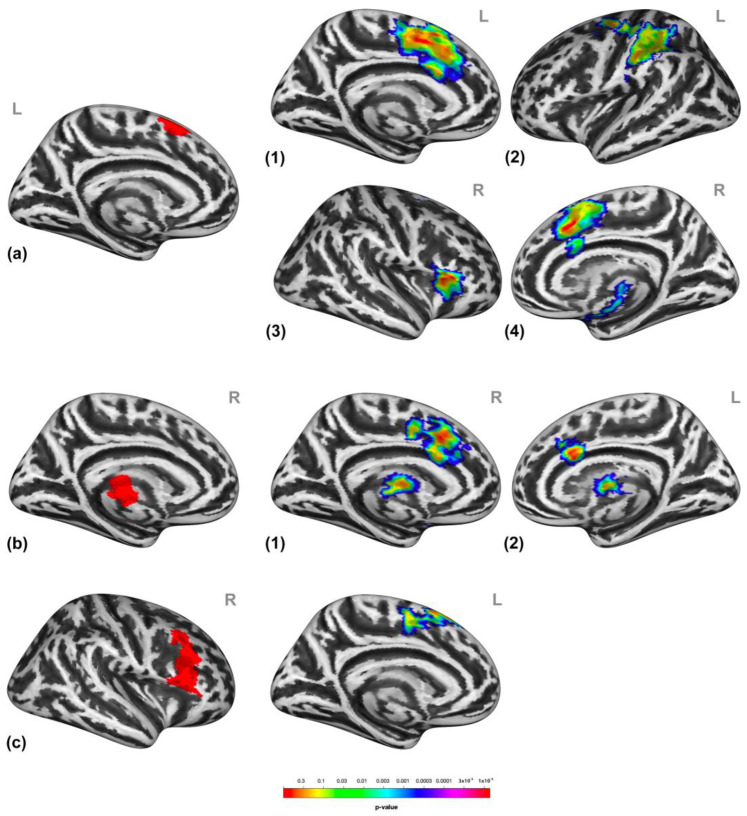
Psychophysiological interactions (PPIs) associated with receiving social victimization compared to prosocial interaction. The functional coupling of the supplementary motor areas (**a**) was increased with the dorsal cingulate cortex, the primary somatosensory cortex (**a1**), the parietal associative cortex (**a2**), the insula (**a3**), the right inferior frontal gyrus, and thalamus (**a4**) during social victimization relative to prosocial interaction. The functional coupling of the thalamus and putamen cluster (**b**) was increased in the thalamus, putamen, and postcentral gyrus (**b1**) as well as with the dorsal cingulate cortex (**b2**) during social victimization relative to prosocial interaction. The functional coupling of the inferior frontal gyrus and the middle frontal gyrus (**c**) was increased with the superior frontal gyrus and the supplementary motor areas (**c**) during social victimization relative to prosocial interaction. All the seeds (**a**–**c**) of the PPIs are highlighted in red. The numbers in the figures on the right indicate the target regions of the PPIs per each seed indicated on the left, respectively. Statistical maps of the PPIs are rendered on an inflated Montreal Neurological Institute template with a cluster-level family-wise error correction of *p* < 0.05. The color bar indicates the *p*-value. L and R indicate the left and right brain hemispheres, respectively.

Furthermore, social victimization increased connectivity between the pre-SMA and the primary somatosensory cortex, the parietal associative cortex, the insula, and the right inferior frontal gyrus and thalamus, as well as between the thalamus and putamen and the thalamus, postcentral gyrus, dorsal cingulate cortex, and the left precentral gyrus, relative to prosocial interaction. Lastly, activation in the right IFG/MFG was associated with increased connectivity with the SFG/SMA (Figure 5) during social victimization relative to prosocial interaction. See Table S2 for detailed cluster information.

### 3.4. Correlations with Psychological Scales

Exploratory analyses showed that brain responses to social victimization were correlated with personality, temperament, and frustration responses in several clusters (see Supplementary Material; Figure S1). In the mFG/SFG, activation was significantly negatively correlated with emotional stability measured using the BFQ (ρ = −0.45, *p* = 0.014); in the pre-SMA, activation was correlated with emotional stability (ρ = −0.37, *p* = 0.042) driven by its control of the emotions subscale (ρ = −0.39, *p* = 0.031), with the irritable (ρ = 0.49, *p* = 0.006) temperament scale of the TEMPS-M, and with the deprivation of positive reinforcement (ρ = 0.42, *p* = 0.022) subscale of the QDF; in the putamen and thalamus, activation was correlated with irritable (ρ = 0.48, *p* = 0.022) scores of the TEMPS-M and with the negative reinforcement (ρ = 0.55, *p* = 0.002) and deprivation of positive reinforcement (ρ = 0.61, *p* < 0.001) subscales of the QDF. Brain activation in the IFG/MFG was correlated with the irritable temperament scale of the TEMPS-M (rho = 0.39, *p* = 0.002). Finally, there were no correlations between reaction times and psychological scales.

## 4. Discussion

In this study, we observed three main findings: First, participants exposed to social victimization displayed higher activation in the left STG/MTG and the bilateral lingual gyrus with reduced connectivity with the contralateral STG/MTG compared to the control condition. Second, when comparing social victimization to prosocial interaction, participants showed higher activations in the bilateral putamen, the thalamus, and the dmMPFC, including bilateral mFG/SFG, pre-SMA, and the right IFG/MFG, as well as increased connectivity in the salience network. Third, brain activation in the dmPFC, putamen, and striatum during social victimization was correlated with affective temperaments and reduced tolerance to frustration.

STG and MTG are active during social processes [28], including attention, emotional regulation, empathy, and ToM [29,30,31]. Altered activation of these regions has been reported in social exclusion [32]. Taken together, these results suggest that a functional reorganization of the ToM network may be necessary to convey the negative meaning of the situation when exposed to social victimization [33]. Furthermore, we observed a hyperactivation of the lingual gyrus in social victimization, possibly related to a bottom-up mechanism aimed at a better understanding of the situation, as a first step in processing the interaction and its social valence [18].

Additionally, increased activation of the thalamus and putamen was observed during the processing of social victimization, which was associated with irritable temperament, intolerance to frustration, and punishment. The thalamus is involved in emotion processing [34] and is activated when we perceive social interaction scenes, regardless of emotional valence [35]. The putamen also plays an important role in the social context, being part of the “hate circuit”, a set of brain regions that activate in response to a hated face and may be related to the preparation, execution, and withholding of motor action in response to this negative emotion [36]. Taken together, these results could suggest that thalamo-putaminal hyperactivation is associated with emotional instability and dysregulation, a common characteristic of irritable temperament, which can contribute to triggering stronger emotional activation when exposed to antisocial behaviors. In addition, we speculate that these brain areas may mediate frustration in response to socially stressful situations, and this response may be exaggerated in subjects prone to mood dysregulation. Furthermore, when presented with antisocial behaviors, the thalamus and putamen showed increased functional coupling with the thalamus itself, insula, cingulate cortex, primary sensorimotor cortex, parietal associative cortex, and nodes of the salience network.

We also found an engagement of the dmPFC (mFG/SFG and pre-SMA). Previous studies have shown that the dmPFC is associated with social processing [37], with a specific role in understanding others’ intentions [38], and promoting emotional upregulation in social situations via reappraisal [39]. The mFG/SFG is an area of convergence between the dorsal and ventral attention networks [40], serving as a circuit breaker to interrupt endogenous attentional processes in the dorsal network and reorient attention to an exogenous stimulus [41]. Interestingly, the mFG/SFG is activated during the suppression of socially unacceptable responses [42]. In our exploratory analysis, participants who presented higher scores on the conscientiousness personality trait (see Supplementary Material) also showed greater activation in the mFG/SFG. This is probably related to prioritized attention and greater self-awareness in social situations that careful individuals tend to exhibit. Additionally, the mFG/SFG activity was negatively correlated with emotional stability and positively with irritable temperaments, which is in line with previous studies showing an association between dmPFC volume and irritability [43]. We also found that activation in the mFG/SFG was positively correlated with reduced tolerance to frustration in response to deprivation of reward, supporting the role of this region in emotional regulation associated with social interactions. The pre-SMA is involved in motor planning and response inhibition in cognitively conflicting situations [44]. We hypothesize that this region may play a role in the behavior associated with antisocial interactions. Indeed, pre-SMA activity was positively correlated with neuroticism and irritable temperament, which is characterized by increased motor and emotional activity. Notably, we found that the processing of social victimization was associated with increased connectivity of the pre-SMA with the insula/IFG, dorsal cingulate cortex, somatosensory cortex, putamen, and thalamus, critical nodes of the pain matrix [45] that are also capable of detecting the salience of a stimulus [46]. Indeed, social pain threatens the integrity of an individual’s demand for basic social needs [9], requiring a behavioral action aimed at restoring social integrity.

Finally, we observed right IFG hyperactivation when participants were exposed to social victimization. The IFG is an area related to executive functions and response inhibition [47], which is activated in response inhibition tasks, such as the stop-signal task [48]. Remarkably, we found that this area showed a greater functional coupling with the pre-SMA/SMA during social victimization, which might suggest a role of the IFG in controlling the behavioral response. Additionally, participants with irritable personality traits showed greater activation of IFG, suggesting inefficient processing of response inhibitory control.

Although our study was not designed to explore the temporal dynamics of brain responses to antisocial behaviors, our findings might provide initial evidence to expand the framework of the need-threat temporal model of ostracism to social victimization [9]. We speculate that at first, the ToM network and bottom-up occipital activation could be recruited to detect antisocial behavior, and altered coupling may contribute to signaling this unexpected social behavior (minimal signal stage). Then, subcortical areas, such as the thalamus and putamen, might immediately process social interaction and its negative emotional impact on the recipient (reflexive stage). Subcortical activation is then followed by activity in the dmPFC, which could be involved in understanding others’ intentions in this conflicting situation and preparing a response to others’ antisocial behavior. This activity is then associated with the activation of more frontal areas, which might potentially be recruited to regulate the response and the emotional impact elicited by interaction with someone who performs an antisocial behavior (reflective stage). Interestingly, we observed that this cascade of events is modulated by aspects of temperament and personality so that individuals with lower emotional stability and with irritable temperamental traits show greater brain responses in these regions. The type of response to frustrating events was also correlated with brain activity, and individuals with greater susceptibility to frustrating situations recruited these areas more strongly.

Although previous studies have investigated the neural correlates of negative social interactions, such as social exclusion [49], this is, to the best of our knowledge, the first fMRI study that specifically addresses the neural correlates of social victimization. Here, we show for the first time that the processing of social victimization seems to be based on a set of regions associated with salience, emotional processing, and regulation. Our study shows convergent results and supports the functional network underlying social victimization previously suggested in psychiatric patients. However, we must also acknowledge some limitations. First, our paradigm did not allow us to estimate the temporal order of engagement of the neural areas involved in receiving antisocial behavior. The phenomena that are activated during the processing of these complex scenarios are not amenable to be temporally modeled, and we chose to maximize the power to detect significant differences across real-world social conditions rather than estimating event-related responses to non-natural conditions. The brain responses to the interactions are not bidirectional and, therefore, limited in the interactivity between individuals that characterizes social exchanges. Nonetheless, this design allowed us to identify direct brain responses to social victimization, without the confounder of reactive behavior that can follow the exposure to antisocial behavior. Lastly, in our study, we recruited only young adults of both sexes. Age and sex can also affect social victimization and, ultimately, the neural correlates of this process [50]. These are important areas of research for the field that can be developed in future studies.

## 5. Conclusions

In conclusion, our findings suggest that social victimization is associated with a widespread network of cortical and subcortical activity aimed at detecting, responding, and restoring the social integrity of an individual. If the situation persists, a resignation stage can follow with a sense of unworthiness, alienation, helplessness, and, ultimately, depression. Future studies involving the neurobiology of the long-term consequences of persistent social victimization, i.e., bullyism, are warranted.

## Data Availability

The data that support the findings of this study are available on reasonable request from the corresponding author.

## Supplementary Materials

The following supporting information can be downloaded at: https://www.mdpi.com/article/10.3390/brainsci13030474/s1. Figure S1. Scatterplots of brain activation and temperament and frustration scores. Brain responses in the thalamus-putamen (A) and right inferior/middle frontal gyrus (B) during social victimization relative to prosocial interaction correlated with hyperthymic and irritable temperament scores measured with the TEMPS-M scale, respectively. Brain responses in thalamus-putamen (C) during antisocial responses relative to prosocial responses were correlated with frustration with negative reinforcement measured using QDF. Activation responses are measured in arbitrary units. QDF, questionnaire of daily frustration. Table S1. Confusion matrix showing the affective responses relative to the type of social stimulus. Table S2. Summary of peak coordinates, significance, anatomical label and PPI-seed for significant clusters of brain activation and psychophysiological interactions (PPI) during social victimization.
