# Supplementary material for: Neural Correlates of Antisocial Behavior: The Victim’s Perspective
Source: Brain Sci. 2023 Mar 10;13(3):474. doi: 10.3390/brainsci13030474 (PMC10045997; doi:10.3390/brainsci13030474)
Supplement: Supplementary file 1 [file brainsci-13-00474-s001.zip › brainsci-2216975-supplementary.pdf]

Supplementary Material

Neural Correlates of Antisocial Behavior: the Victim's Perspective

Nicolò Trevisan [1,2], Giulia Cattarinussi [1,2], Daniele Olivo [1], Andrea Di Ciano [3], Lucia Giudetti [3], Alan Pampallona [3,†], Katharina M. Kubera [4], Dusan Hirjak [5], Robert Christian Wolf [4] and Fabio Sambataro [1,2,*]

[1] Department of Neuroscience (DNS), University of Padua, 35121 Padua, Italy; trevisannicolo@gmail.com (N.T.); giulia.cattarinussi@unipd.it (G.C.); daniele.olivo91@gmail.com (D.O.)
[2] Padua Neuroscience Center, University of Padua, 35129 Padua, Italy
[3] Fondazione Giancarlo Quarta, 20129 Milan, Italy; andrea.diciano@fondazionegiancarloquarta.it (A.D.C.); lucia.giudetti@fondazionegiancarloquarta.it (L.G.)
[4] Center for Psychosocial Medicine, Department of General Psychiatry, Heidelberg University, 69115 Heidelberg, Germany; katharina.kubera@med.uni-heidelberg.de (K.M.K.); christian.wolf@med.uni-heidelberg.de (R.C.W.)
[5] Department of Psychiatry and Psychotherapy, Central Institute of Mental Health, Medical Faculty Mannheim, Heidelberg University, 68159 Mannheim, Germany; dusan.hirjak@zi-mannheim.de
* Correspondence: fabio.sambataro@unipd.it
† Deceased.

Material and Methods

Subjects. A total of 30 young healthy Italian subjects (17 females; age range between 21 and 30 years) with normal or corrected-to-normal vision participated in the study. Exclusion criteria were: a) current or past medical, psychiatric, or neurological disease; b) history of substance abuse or dependence (excluding nicotine); d) cognitive impairment; e) head trauma with loss of consciousness; f) contraindications to magnetic resonance imaging. Participants were excluded from the study if they exhibited excessive head motion, defined as greater than 3.0 mm or 3.0 degrees of rotation during the scanning session. Written informed consent was obtained from all participants after a detailed explanation of all aims and procedures of the study, which was conducted in accordance with the principles expressed in the Declaration of Helsinki and approved by the Institutional Review Board of the University of Parma (Italy, protocol ID: UNIPRMR750v1)

Social Interaction Task. Each participant performed a social interaction task during the fMRI scan (Figure 1). Each trial consisted of the presentation of different scenes of social interactions, each followed by an affect rating task. Participants were presented with a sequence of three vignettes and three sentences in between, depicting a dyadic social interaction. The vignettes included an everyday interaction scene between two characters: the recipient (portrayed with gray hair), with whom the participants were requested to identify, and the agent, the person interacting with the recipient. In between the vignettes, sentences meant to complement the vignettes appeared on the screen to help empathize. One type of social interaction was displayed: social victimization, prosocial, and control conditions. During each condition, the recipient performed a task or received feedback on an action that he/she performed. In the prosocial condition, the recipient was helped perform a task or given positive feedback by the agent. In the social victimization condition, the recipient was ignored during a task that needed external help or was given harsh feedback. The control condition had visual and conceptual characteristics similar to those of the other social interaction conditions and showed a scene where the receiver accomplished a result without the direct involvement of the agent. Due to our focus on social victimization interactions, participants were shown 60 social victimization, 30 prosocial, and 30 control scenes without repetitions. After the social interaction scene, an affect rating image comprising three smileys with happy, neutral, and sad faces, and instructions to rate his/her own affect following the last social interaction by pressing a button on an MRI-compatible response device was displayed to the participant.

Scenarios and stimuli. Suprathreshold visual complexity metric (R_{spt})[1] and the sentence length were compared across helping conditions to measure visual complexity (for pictures) and textual complexity (for sentences), respectively. Briefly, the R_{spt} is based on detectability suprathreshold, as it quantifies the number of detectable regions in an image and considers an image with a high number of regions to be more complex[1]. We did not find any difference (all p's>0.05) between social conditions in visual complexity, (R_{spt} index, mean ± SD: Antisocial condition= 101.1 ± 44.1; Prosocial condition= 118.5 ± 42.8; Control condition= 114.8 ± 42.1) and number words (mean ± SD: Antisocial condition= 12.9 ± 3.3; Prosocial condition= 11.8 ± 2.7; Control condition= 11.9 ± 3.4) of the vignettes.

Across all the participants, the antisocial vignettes were associated with a negative rating the 59.36% of the times, the control vignettes were judged as a neutral rating for 53.04% of the ratings while the prosocial vignettes were assigned a positive rating for the 87.83% of the time.

Neuropsychological evaluation. Subjects performed a self-administered neuropsychological evaluation before performing the task in the scanner. Personality traits were assessed with the Big Five Questionnaire (BFQ)[2], a 132-item questionnaire aimed at measuring personality traits according to the five-factor theory of personality, including neuroticism, extroversion, agreeableness, conscientiousness, and openness to experience. Affective temperaments that are believed to measure the biological component, mainly genetic, of personality traits, include cyclothymic, hyperthymic, irritable, depressive, and anxious dimensions were evaluated with the Italian version of the 35-item Temperament Evaluation in Memphis, Pisa, and San Diego (TEMPS-M)[3]. Lastly, reactions to frustrating events were assessed using the Questionnaire of Daily

Frustrations (QDF)[4], a questionnaire depicting 32 frustrating situations including responses to deprivation of rewards and confrontation with punishments.

Image acquisition. MRI scans were acquired at the Local Health Unit of Parma on a 3 Tesla GE Discovery MR750scanner equipped with a 16-channel multi-array head-coil. High-resolution T1-weighted images were obtained using magnetization-prepared rapid acquisition gradient-echo sequence (MPRAGE) with a time of repetition/time of echo= 9700 ms/3.97 ms, field of view= 256×256 mm^2, and a voxel size= 0.5×0.5×0.9 mm^3. Functional data were obtained using a gradient-echo-planar imaging sequence with the following parameters: time of repetition/time of echo = 2000 ms/ 30 ms, flip angle= 90°, field of view= 240×240 mm^2, and a voxel size= 3.2×3.2×3.5 mm^3. The social interaction task scan, which included the same number of trials per condition across runs, comprised 338 volumes per run, with a total of 4 runs.

Imaging analysis. Pre-processing was performed with SPM12 (https://www.fil.ion.ucl.ac.uk/spm/software/spm12/). First, we checked the quality of the MRI data by visually inspecting each image to ensure that there were no artifacts or lesions. The images were then reoriented, realigned to correct for head motion, and coregistered to the individual structural data. Structural scans were segmented using DARTEL in SPM12. Then, the parameters estimated from the normalization of gray matter images into the Montreal Neurological Institute standard space were applied to functional images. A 6-mm full width at half maximum Gaussian kernel was applied to smooth normalized images that were analyzed using a general linear model within SPM12. The task was modeled as a block-design with four regressors for task conditions [three regressors for the whole social interaction condition (prosocial, social victimization, and control) and one for the affective rating task], and 6 nuisance covariates (motion parameters). For each condition, a box-car was convolved with a canonical hemodynamic function. In the first-level analyses, whole-brain contrast maps of direct comparisons between social interaction conditions were calculated for each individual subject. Individual contrasts were then entered into a random-effects one-sample t-test analysis for each contrast. Psychophysiological interaction (PPI) analyses were performed to identify social task condition-dependent changes in connectivity between the clusters of increased activation during social victimization processing and the whole-brain: lingual gyrus, left superior and middle temporal gyrus (STG/MTG), medial and right superior frontal gyrus (SFG), pre-supplementary motor area (pre-SMA), the right inferior/middle frontal gyrus (IFG/MFG), the putamen and the thalamus. The physiological regressors were calculated as the first eigenvariates from significant clusters at the group level, adjusted for motion, mean-centered, filtered, and deconvolved. A model including the psychological regressors (t-contrasts for social victimization>neutral behavior and social victimization>prosocial behavior, respectively), the physiological regressors, and their interaction (cross-product of the psychological and the physiological regressors) was calculated for each subject. Positive and negative interaction terms were then analyzed at the second level using one-sample t-tests, respectively. For all second-level analyses, we applied a $p<0.05$ family-wise error correction at the cluster level with a cluster-defining threshold of uncorrected $p<0.001$ at the voxel level. No participants met any of the exclusion criteria, therefore all the subjects were included in the fMRI analysis.

Behavioral analyses. Reaction times were analyzed using repeated-measures ANOVA between task conditions, followed by pairwise contrasts controlled for multiple comparisons with a Bonferroni correction. Unfortunately, due to a computer glitch, behavioral data from only 15 participants were available. Brain-behavior correlations between the first eigenvariate of each significant cluster from significant social victimization>prosocial behavior contrasts and neuropsychological measures were performed using Spearman's correlations, due to the non-normal distribution of the variables. For these analyses, the significance was set at $p<0.05$. All analyses were performed using Jamovi (https://www.jamovi.org/).

Exploratory correlations.
We explored the correlation between brain activation and BFQ scales and temperament traits of TEMPS-M that were not hypothesized in our study. Brain activation during social victimization relative to prosocial

behavior in the right lingual gyrus correlated with the consciousness trait of the BFQ (rho=0.375, p=0.041) - and this result was driven by scrupulousness subscale (rho=0.462, p=0.009) – as well as activation in the putamen and thalamus was correlated with the cyclothymic (rho=0.362, p=0.049) and hyperthymic (rho=0.427, p=0.019) temperament scores. Finally, activation of the pre-SMA during social victimization was correlated with hyperthymic temperament (rho=0.376, p=0.041).

Supplementary Figures

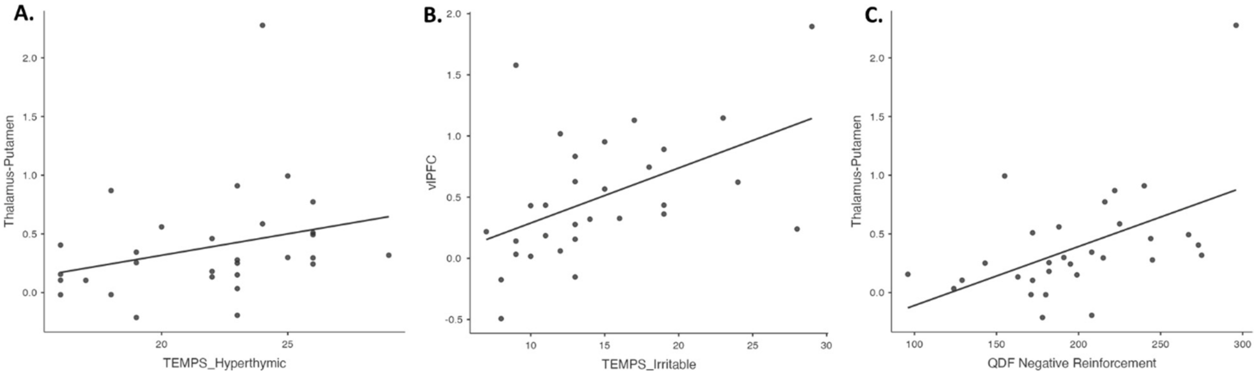

Figure S1. Scatterplots of brain activation and temperament and frustration scores. Brain responses in the thalamus-putamen (A) and right inferior/middle frontal gyrus (B) during social victimization relative to prosocial interaction correlated with hyperthymic and irritable temperament scores measured with the TEMPS-M scale, respectively. Brain responses in thalamus-putamen (C) during antisocial responses relative to prosocial responses were correlated with frustration with negative reinforcement measured using QDF. Activation responses are measured in arbitrary units. QDF, questionnaire of daily frustration

Supplementary Tables

Table S1. Confusion matrix showing the affective responses relative to the type of social stimulus

	**Affective response**		
**Condition**	Negative	Control	Positive
Social victimization	59.36%	38.36%	2.27%
Control	32.95%	53.04%	13.99%
Prosocial	2.25%	9.90%	87.83%

Table S2. Summary of peak coordinates, significance, anatomical label and PPI-seed for significant clusters of brain activation and psychophysiological interactions (PPI) during social victimization

Coordinates (x/y/z)	Cluster Size (k)	p-value	Anatomical Region	PPI seed
**Brain activation**				
Social victimization > control				
-54/-36/0	119	0.028	Middle/Superior temporal gyrus	
12/-78/6	242	0.001	Lingual Gyrus	
Social victimization > prosocial interaction				
3/48/33	239	0.002	Dorsomedial prefrontal cortex	
-6/18/60	168	0.009	Pre-supplementary Motor Area (Pre-SMA)	
0/-21/0	389	0.001	Thalamus/Putamen	
45/21/6	233	0.002	Inferior/Middle/Superior frontal gyrus (IFG/MFG/SFG)	
**Psychophysiological interactions (PPI)**				
Social victimization < control				
-9/27/57	226	0.001	SFG/SMA	IFG/MFG/SFG
Social victimization > prosocial interaction				
-12/6/45	657	0.001	Cingulate Gyrus	Pre-SMA
-42/-39/60	504	0.001	Postcentral Gyrus	Pre-SMA
36/21/12	252	0.001	IFG	Pre-SMA
-12/-6/-9	160	0.012	Insula	Pre-SMA
-45/-3/9	117	0.003	Putamen	Pre-SMA
-3/-18/9	243	0.001	Thalamus	Thalamus/Putamen
3/18/24	171	0.001	Cingulate Gyrus	Thalamus/Putamen
-30/-12/63	141	0.004	Precentral Gyrus	Thalamus/Putamen
